# Differences in how NMDA antagonists modulate negative affective biases in male rats may serve as a predictor of clinical efficacy in major depressive disorder

**DOI:** 10.1038/s41398-026-04133-z

**Published:** 2026-05-29

**Authors:** Justyna K. Hinchcliffe, Katie Kamenish, Julia Bartlett, Roberto Arban, Bastian Hengerer, Emma S. J. Robinson

**Affiliations:** 1 University of Bristol, School of Physiology, Pharmacology and Neuroscience, Biomedical Sciences Building, Bristol, BS8 1TD UK; 2 CNS Diseases Research, Boehringer Ingelheim GmbH & Co. KG, Biberach an der Riss, Germany

**Keywords:** Learning and memory, Depression

## Abstract

Affective biases shape cognitive and emotional behaviour and are important in major depressive disorder (MDD). Modulation of affective biases by the NMDA antagonist, ketamine, may underlie its antidepressant effects but not all NMDA antagonists are efficacious. Some studies suggest ketamine’s efficacy involves non-NMDA mechanisms e.g. via its active metabolite HNK (2R, 6R)-hydroxynorketamine), but an alternative hypothesis is that pharmacodynamic differences relating to ion trapping and/or affinity may be relevant. This study used a rat model of affective biases to investigate how different NMDA antagonists influence retrieval of a negatively biased memory, both acutely (<60 min) and ~24 h post-treatment. We compared compounds tested clinically (lanicemine, memantine, CP101,606), reference antagonists with different pharmacodynamic profiles (phencyclidine, PCP; ephenidine), and HNK. PCP, lanicemine, and ephenidine but not CP101,606 acutely attenuated negative biases. At 24 h, these effects were sustained for CP101,606 and ephenidine with a trend towards inducing a positive bias. Lanicemine’s effects were sustained only at high doses and PCP and memantine had no effects. HNK looked like ketamine but only at doses higher than those achieved through metabolism of the effective ketamine dose. These findings suggest that sustained modulation of affective biases, particularly when the treatment facilitates re-learning with a more positive affective valence, correlates with therapeutic efficacy. Based on preliminary findings using reference NMDA antagonists, differences in antidepressant efficacy may relate to ion trapping properties. Very high or very low ion trapping appear less effective than intermediate compounds like ketamine and ephenidine or subunit-selective antagonists like CP101,606.

## Introduction

The term affective bias is used to describe how affective states influence information processing and are thought to play an important role in major depressive disorder (MDD) [1, 2]. Different cognitive domains have been shown to be modulated by positive or negative affective states including learning and memory, decision-making and attention [3, 4]. In MDD these affective biases change perception, memory retrieval and decision-making amplifying negative emotions while diminishing positive ones [3, 4]. Negative affective biases are hypothesised to play an important role in the development and perpetuation of mood disorders [1–3] and, modulation of negative affective biases has been shown with conventional delayed onset antidepressants in both humans [1] and rats [5–7], and rapid acting antidepressants (RAADs) in rats [7, 8]. There is mounting evidence to suggest affective biases represent a ‘behavioural biomarker’ of depression. They are observed in patients and at-risk individuals [1, 2, 9] and develop in a rodent model of early life stress [10]. Affective biases can be objectively measured using behavioural tasks with evidence that changes in patients with depression correlate with the core symptoms of low mood and loss of interest in rewarding activities [11–13]. Studies using acute doses of conventional antidepressants in healthy volunteers and patients suggests early modulation of affective biases could predict antidepressant response and may drive clinical changes through effects on experience-dependent learning and memory [1, 2, 14].

Findings in our rat model of negative affective biases suggests the NMDA antagonist, ketamine, and other RAADs, such as the psychedelic, psilocybin, modulate negative affective biases via mechanisms distinct from those seen with conventional antidepressants [8]. Conventional antidepressants modify new experiences meaning there is a delay in subjective effects on mood while these positively biased experiences generate new memories. In contrast, our rodent data suggests the ability of RAADs to modulate affective biases associated with past experiences could explain their rapid and sustained antidepressant effects [8].

Since the first clinical study reporting a rapid and sustained antidepressant effect with ketamine [15], clinical trials and rodent studies have explored the potential efficacy of other NMDA antagonists. Ketamine’s clinical benefits in MDD are observed within hours of acute administration and can last for up to 14 days [15, 16]. However, studies with other NMDA antagonists, and even different doses of ketamine, have found mixed results in patients suggesting all NMDA antagonists are not similarly efficacious [16–20]. Different hypotheses have been proposed to explain this lack of efficacy including ketamine acting through non-NMDA mechanisms e.g. via its metabolite hydroxynorketamine or through opioid receptors [21, 22]. NMDA receptors are ligand-gated ion changes formed from different sub-units (NR2A, 2B, 2C and 2D) with regional and cellular distributions that influences their functional effects [23]. Trapping channel blockers permit agonist dissociation and channel closure while remaining bound within the channel pore [24]. Therefore, it is predicted that the greater the trapping blockade, the greater the potency and psychotomimetic effects. The extent of trapping depends on drug affinity, kinetics of channel opening/closing, and the compound’s pKa value and lipophilicity [25]. Very high ion trapping, as seen with PCP, can lead to excessive accumulation and prolonged receptor blockade [26]. By contrast, compounds with low ion trapping and low affinity, such as memantine and lanicemine, tend to dissociate too quickly to produce sustained modulation of glutamatergic signalling and lack antidepressant efficacy [19, 25]. Ketamine has with moderate to high ion trapping and is only effective as a RAAD at low doses [25]. Both ketamine and a pharmacodynamically similar NMDA antagonist, ephenidine are also used recreationally and in patients with substance use disorder [27–29]. These pharmacodynamic and clinical differences are not reflected in studies involving conventional animal models of MDD such as the forced swim test [30] limited the potential to use these methods to reliably predict antidepressant efficacy.

In this study, we used a rat model of negative affective biases to investigate the effects of different NMDA antagonists as well as the ketamine metabolite, HNK. We have previously shown that low but not high dose ketamine selectively modulates affective biases in the rat affective bias test (ABT) [8]. We also found ketamine and the NR2B selective antagonists CP101,606, modulate affective biases and decision-making behaviour [31]. We localised these effects to the medial prefrontal cortex [7, 8] and in the ABT, we show that the sustained effects of ketamine involve experience-dependent plasticity with negatively biased memories re-learnt post-treatment with a more positive affective valence [8]. To investigate the acute versus sustained modulation of negative affective biases, we first generated a negatively biased memory then administered the treatment either < 1 h or ~24 h before a memory retrieval test [32]. We also tested any treatment which induced a significant acute modulation of affective biases in the control reward learning assay (RLA) so we could determine if the effects were specific. We first tested if the ketamine metabolite (2*R*, 6*R*)-hydroxynorketamine replicated the effects of ketamine in the ABT which would support a non-NMDA mechanism underlying ketamine RAAD effects [22, 33]. We next compared the effects of NMDA antagonists which have failed in clinical trials for MDD and/or have different ion trapping properties and affinity at the NMDA receptor. To further explore the hypothesis that ion trapping properties impact on affective bias modification, we also tested ephenidine (two ringed *N*-ethyl-1,2-diphenylethylamine) which has similar pharmacodynamic properties as ketamine but lacks affinity for opioid receptors and does not produce the metabolite HNK. The final compound tested was CP101,606, the GluN2B subunit selective NMDAR antagonist that has demonstrated promising rapid acting antidepressant effects in human clinical trials [17] but failed to progress due to toxicity issues.

## Materials and methods

### Animals

Seven separate cohorts of male Lister Hooded rats (Envigo, UK) were used in these experiments (*n* = 10–12 per group; Table S1 and Figure S1). Male rats weighed approximately 300–330 g (10–11 weeks old) at the start of experimental manipulations. This study utilized only male rats; however, previous research in female rats and different rat strains showed a very consistent picture in terms of both positive and negative affective bias modification across both pharmacological and psychosocial manipulations of affective state [5]. The sample size was based on our previous affective bias test studies and a meta-analysis which suggested a medium to large effect size for the drug-induced negative bias and reward-induced bias in Lister Hooded rats [5, 34]. All animals were pair-housed in standard enriched laboratory cages (55 × 35 × 21 cm) with woodchip, paper bedding, cotton rope, wood chew, cardboard tube and red Perspex house (30 × 17 × 10 cm), under a 12:12 h reverse light-dark cycle (lights off at 08:00 h) and in temperature-controlled conditions (21 ± 1 °C). Rats were food restricted to approximately 90% of their free-feeding weights matched to the normal growth curve [~18 g of food per rat/day laboratory chow (Purina, UK)] and were provided with water *ad libitum*. The behavioural procedures and testing were performed during the animals’ active phase between 09:00 and 17:00 h. All experimental procedures were conducted under the UK Animals (Scientific Procedures) Act 1986 and were approved by the University of Bristol Animal Welfare and Ethical Review Body and UK Home Office (PPL PP9516065).

### Affective bias test training and testing

The testing apparatus and pre-training handling and training protocol followed that of Hinchcliffe & Robinson [32]. Training involved animals learning to dig in ceramic bowls containing sawdust over 5 days with increasing levels of difficulty. The final session tested their ability to learn the task rule in a novel discrimination session where rats had to dig in the correct digging substrate to finding a food reward. Choice of the reward-paired substrate was marked as a ‘correct’ trial, digging in the unrewarded substrate was classified as an ‘incorrect’ trial and if an animal failed to approach and explore the bowls within 10 s, the trial was recorded to be an ‘omission’. Trials were continued until the rat achieved six consecutive correct choices for the reward-paired substrate. The discrimination session was used to confirm that the animals could achieve our learning criterion of six consecutive correct trials in less than 20 trials. Once animals successfully reached criteria in the discrimination session, they were considered trained. All animals then progressed to a reward learning assay protocol to confirm that they would exhibit a reward-induced bias and were therefore performing the task correctly and making their choice based on the memory associated with the digging substrate.

**Fig. 1:**
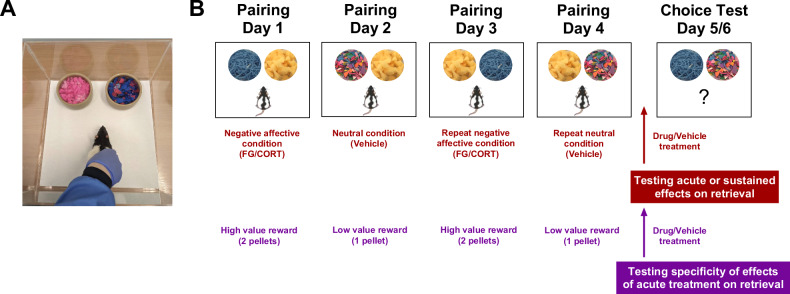
The affective bias test and reward learning assay. Photograph illustrating the arena with bowls and rat position (**A**). Overview of the affective bias test and reward learning assay protocol (**B**). FG: FG7142, CORT: corticosterone. Rat graphic generated by open access AI software.

During testing, each week consisted of four consecutive pairing sessions to generate two independent cue-specific experiences. Using a within-subject design, each animal learnt a specific substrate-reward association under either a control or treatment conditions. A choice test on the fifth (acute modulation or reward learning assay) or sixth (sustained modulation) day of the same week assessed memory retrieval with or without drug pre-treatment (Fig. 1). Details of all pairing sessions and choice test procedures are explained in the supplementary methods (Tables S2A and S2B) and experimental timeline for all cohorts presented in Figure S1. All drug treatments, pairing substrates and order of presentation were fully randomised in all studies. Affective biases generated using this protocol were quantified during the choice test when the two previously rewarded substrates (‘A’ and ‘B’) were presented at the same time over 30 randomly reinforced trials (for details, see [32]). The animals’ choices and latency to dig were recorded.

### Control reward learning assay

To assess the specificity of the acute effects on retrieval, the same treatments were tested in the reward learning assay. The RLA follows a similar protocol to the ABT with animals experiencing two independent substrate-reward associations over 4 pairing sessions (Fig. 1) with a choice test on the 5th day however, a reward-induced bias is generated by pairing the cues with either a high (2 pellet) or low (1 pellet) reward outcome. No treatments are given during the pairing sessions but acute pre-treatments can be used before the choice test to determine if a drug has any effects on learning and memory which are not related to affective bias modification. Treatments that had no effect in the RLA are considered to selectively modulate affective state-induced biases, whereas treatments that impair RLA performance are interpreted as producing general, non-specific effects on memory retrieval. The RLA was only used for treatments where acute effects had been observed in the ABT.

### Drugs

The drugs used to induce a negative affective bias in rats were corticosterone (10 mg/kg, SC) and FG7142, benzodiazepine inverse agonist (3 mg/kg, SC). The NMDA receptor antagonists tested were HNK (0.3, 1, 3 mg/kg, IP, t = −20 min and −24 h), PCP (0.1, 0.3, 1 mg/kg, IP, t = −40 min and −24 h), memantine (0.3, 1, 3 mg/kg, IP, t = −60 min and −24 h), lanicemine (1, 3, 10 mg/kg, IP, t = −60 min and −24 h), CP101,606 (1, 3 mg/kg, IP, t = −60 min and −24 h) and ephenidine (1 mg/kg, IP, t = −60 min and −24 h) (Table S1). All drugs were purchased from Merck (previously Sigma-Aldrich) or MedChemExpress. Ephenidine was kindly provided by Dr Jason Wallach. CP101,606 was dissolved in vehicle of 5% DMSO, 10% cremophor, 85% saline and all remaining NMDA antagonists were dissolved in saline vehicle in a dose volume 1 ml/kg. For corticosterone or FG7142-induced negative affective biases, we selected doses previously reliable in the affective bias test [7, 8]. Although the underlying mechanisms of corticosterone and FG7142 differ, the behavioural outcome is a robust and consistent induction of negative affect [7, 8]. The choice between the use of these compounds in individual experiments is guided primarily by practical considerations. The choice of NMDA antagonists for these studies was based on treatments tested in clinical trials (memantine, lanicemine and CP101,606) and comparing these to an NMDA antagonist known to have high ion trapping properties and potency (PCP). While ideally, these would be aligned with data demonstrating similar levels of receptor occupancy, there is limited pharmacodynamic data from in vivo studies in rats currently available. We therefore chose doses of PCP, memantine, lanicemine and CP101,606 based on previous studies using a judgement bias task which investigated the effects on a related affective bias modulation involving decision-making under ambiguity [31]. HNK doses were based on previous publications [21, 33] and our previous studies with ketamine and an estimate of the concentration of HNK metabolite generated. Ephenidine dose was chosen based on its in vitro pharmacodynamic profile [28] as in vivo bioavailability and occupancy data was not available. Only a single dose was tested as we had limited availability of this compound. Intraperitoneal injection procedures were done using a low-stress, non-restraint method developed in our research group [35]. All animals were habituated to the position required for dosing for five days prior to the experiments. All subcutaneous injections were performed with minimal animal restraint and injected on their left or right flank (changing daily). Each drug was tested as an independent experiment and a within-subject design was used, with the experimenter blind to treatment and with a fully counterbalanced experimental design. Direct comparison between treatments was not made statistically and interpretation was based on the types of affective bias modification observed with each drug rather than any relative effects within the same ABT readout.

### Data analysis

The primary outcome used for the analysis was % choice bias however, analysis of the latency and omission data from the choice test trials were used as a measure of task engagement and sedation. The number of pellets obtained during the choice test was used to check the animals were obtaining the expected 10 pellets from the random reinforcement schedule and not using olfactory cues to identify the reward location. Data were analysed and figures were created using GraphPad Prism 10.2.0 (GraphPad Software, USA). Choice bias score was calculated as the number of choices made for the drug-paired substrate (affective bias test) or two pellets-paired substrate (reward learning assay) divided by the total number of trials multiplied by 100 to give a percentage. A value of 50 was then subtracted to give a score where a choice bias towards the drug-paired substrate gave a positive value and a bias towards the control-paired substrate gave a negative value. Choice bias scores and response latency scores during the choice test were analysed using a repeated measures ANOVA with treatment as the within-subject factor, and as a post-hoc analysis pairwise comparisons were made using a two-tailed paired t-test or Dunnett’s test depending on the number of group comparisons. Individual positive or negative affective biases were also analysed using a one-sample t-test against a null hypothesis mean of 0% choice bias. For each animal, mean trials to criterion and latency to dig during the affective bias test pairing sessions and choice test were analysed using a repeated measures ANOVA with treatment as the factor or a two-tailed paired t-test, with post-hoc pairwise comparisons made using a two-tailed paired t-test comparison between control (vehicle/low reward:1 pellet) and treatment/manipulation (corticosterone/FG7142/high reward: 2 pellets) for each week (drug-induced negative bias retrieval studies and reward learning assay). Analysis of the choice latency and trials to criterion was made to determine the presence of any non-specific effects of treatment, such as sedation. A Shapiro-Wilk test was used to determine a normal distribution for the % choice bias, trials to criterion, and mean latency to dig during pairing sessions and choice test. Mauchly’s sphericity test was used to validate a repeated measures ANOVA. Effect sizes were calculated for the main treatment effects in repeated-measures ANOVAs using partial eta squared (ηp²), and for paired t-tests using Cohen’s *d*. For percentage choice bias across all studies, Cohen’s *d* effect sizes and corresponding confidence intervals were also reported.

## Results

For the memory retrieval studies involving a FG7142 or corticosterone-induced negative bias, six animals that did not exhibit the expected negative bias under vehicle treatment were excluded (see Table S3). Values more than 2 standard deviations from the group mean were also excluded (see Table S3).

**Fig. 2:**
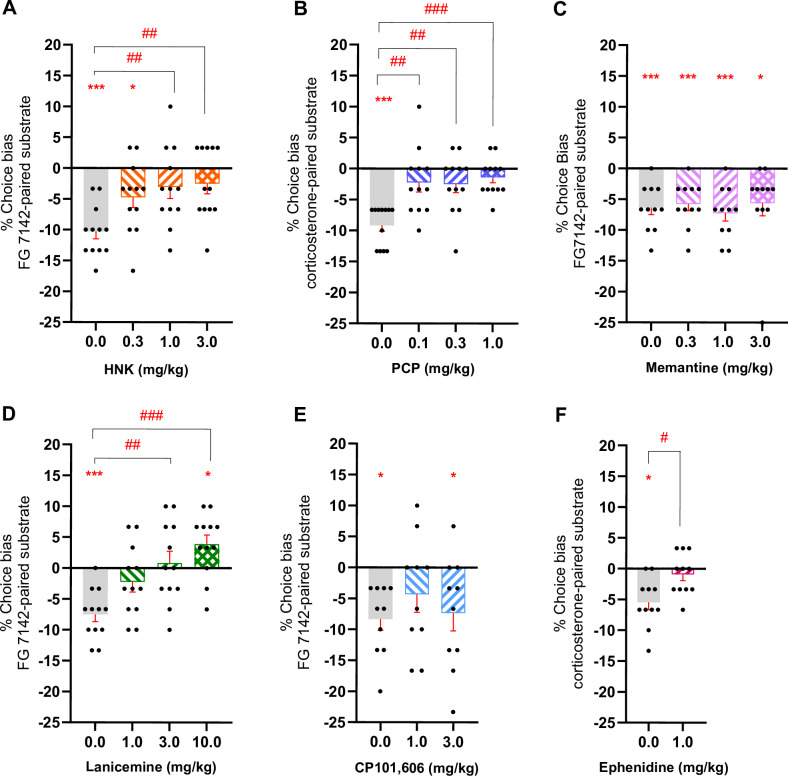
HNK, PCP, lanicemine and ephenidine attenuate negative affective bias in rats. Following induction of a negative affective bias with FG7142 or corticosterone, male rats were injected with HNK (0.3, 1.0, 3.0 mg/kg; t = −20 min; *n* = 12; **A**), PCP (0.1, 0.3, 1.0 mg/kg; t = −60 min; *n* = 12; **B**), memantine (0.3, 1.0, 3.0 mg/kg; t = −60 min; *n* = 11; **C**), lanicemine (1.0, 3.0, 10.0 mg/kg; t = −60 min; *n* = 12, **D**), CP101606 (1.0, 3.0 mg/kg; t = −60 min; *n* = 11, **E**) or ephenidine (1.0 mg/kg; t = −60 min; *n* = 11, **F**) prior to choice test. Only two highest doses of HNK 1.0 and 3.0 mg/kg, all doses of PCP, the middle dose of lanicemine 3.0 mg/kg and one dose of ephenidine 1.0 mg/kg significantly attenuated previously learnt negatively biased memories in rats. The highest dose of lanicemine not only attenuated the negative bias but rats also shown a positive bias at the group level (**D**). Data are shown as mean % choice bias ± SEM (bars) as well as individual data points (dots, *n* = 11–12). Data were analyzed with one sample t-test against a null hypothesis mean of 0% choice bias (*p < 0.05, ***p < 0.001) and pairwise comparisons were done using Dunnett’s test following main effect of treatment in RM ANOVA (*#p* < 0.05, ##*p* < 0.01, ###*p* < 0.001).

In all studies, the control rats made fewer choices for the treatment-paired (either FG7142 or corticosterone-paired) digging substrate consistent with a negatively biased memory (one-sample t-test, *p* < 0.0001, Fig. 2A–F).

Acute HNK at 1.0 and 3.0 mg/kg (repeated measures ANOVA, F3, 33 = 4.861, *p* = 0.0066, *n* = 12, ηp² = 0.306, with Dunnett’s test, *p* < 0.001, Fig. 2A), and PCP at all doses (repeated measures ANOVA, F3, 33 = 7.332, *p* = 0.0007, *n* = 12, ηp² = 0.400, with Dunnett’s test, *p* < 0.001, Fig. 2B), attenuated the negative bias when administered 1 h prior to the choice test. No acute effects were observed following treatment with memantine (Fig. 2C) or CP101,606 (Fig. 2E). The negative bias was also attenuated when animals received the two highest doses 3.0 and 10.0 mg/kg of lanicemine (repeated measures ANOVA, F3,33 = 8.316, *p* = 0.0003, *n* = 12, ηp² = 0.431, with Dunnett’s test, *p* < 0.001, Fig. 2D) and 1.0 mg/kg ephenidine (two-tailed paired t-test: t10 = 3.012, *p* = 0.0131 vs vehicle, *n* = 11, d = 0.908, Fig. 2F). The highest dose of lanicemine not only attenuated the negative bias but rats also showed a positive bias at the group level (one-sample t-test, *p* = 0.0228).

**Fig. 3:**
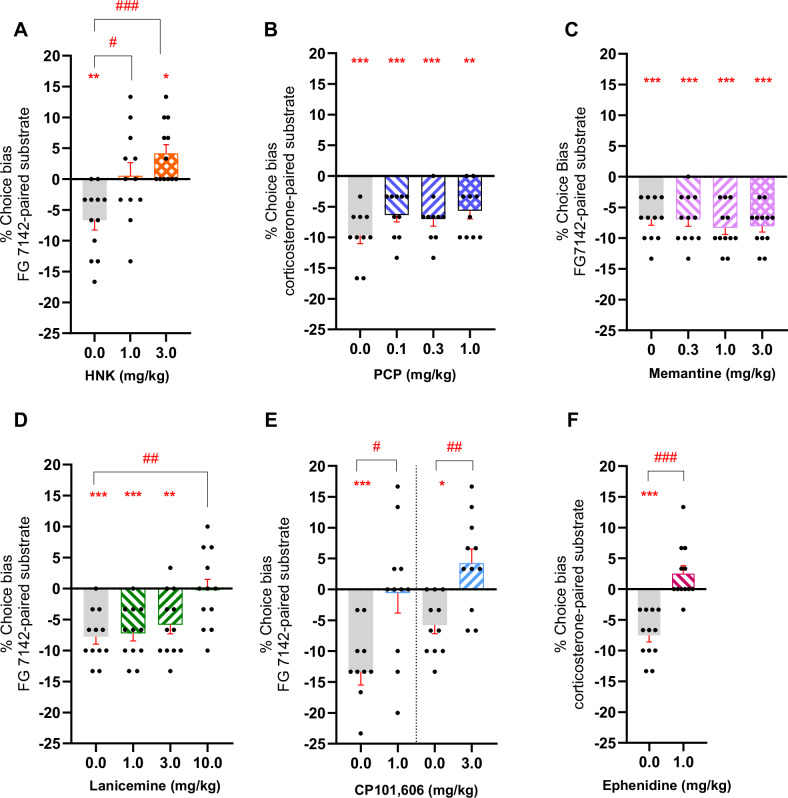
Treatment with HNK, lanicemine, CP101606 and ephenidine at 24 h prior to choice test resulted in sustained attenuation of negative affective bias in rats. Following FG7142- or corticosterone-induced negative bias rats were treated with HNK (0.3, 1.0, 3.0 mg/kg; *n* = 12; **A**), PCP (0.1, 0.3, 1.0 mg/kg; *n* = 10; **B**), memantine (0.3, 1.0, 3.0 mg/kg; *n* = 12; **C**), lanicemine (1.0, 3.0, 10.0 mg/kg; *n* = 12, **D**), CP101606 (1.0, 3.0 mg/kg; *n* = 11, **E**) or ephenidine (1.0 mg/kg; *n* = 12, **F**) 24 h before the choice test. Low dose of HNK 1.0 mg/kg, the highest dose of lanicemine 10.0 mg/kg, both doses of CP101606 and ephenidine 1.0 mg/kg significantly blocked the development of negative biases in rats, indication sustained attenuation similar to acute effects. Only the highest dose of HNK at 3.0 mg/kg significantly inversed the bias into a positively valenced (**A**), similarly to the effects observed following ketamine treatment in Hinchcliffe et al. [8]. Data are shown as mean % choice bias ± SEM (bars) as well as individual data points (dots, *n* = 10–12). Data were analyzed with one sample t-test against a null hypothesis mean of 0% choice bias (*p < 0.05, **p < 0.01, ***p < 0.001) and pairwise comparisons were done using Dunnett’s test following main effect of treatment in RM ANOVA (#*p* < 0.05, ##*p* < 0.01, ###*p* < 0.001).

In the protocol testing modulation of a negative affective bias 24 h after treatment, the highest dose of HNK led to a positive affective bias in this test indicating re-learning with a more positive affective valence (one sample t-test: t11 = 2.916, *p* = 0.0140, *n* = 12) (Fig. 3A). HNK treatment at the lower dose of 1 mg/kg (*n* = 12, with Dunnett’s test, *p* < 0.05, Fig. 3A) resulted in a sustained attenuation of the negative bias. PCP (Fig. 3B) and memantine (Fig. 3C) had no effect at 24 h post treatment with all groups having similar negative affective biases. The highest dose of lanicemine (repeated measures ANOVA, F3,33 = 6.099, *p* = 0.0020, *n* = 12, ηp² = 0.389, with Dunnett’s test, *p* < 0.001, Fig. 3D) and both CP101,606 doses (1 mg/kg: two-tailed paired t-test: t10 = 2.753, *p* = 0.0204, *n* = 11, d = 0.830, Fig. 3E, and 3 mg/kg: two-tailed paired t-test: t10 = 3.816, *p* = 0.0034, *n* = 11, d = 1.151, Fig. 3E) as well as ephenidine (two-tailed paired t-test: t11 = 5.196, *p* = 0.0003, *n* = 12, d = 1.500, with Dunnett’s test, *p* < 0.001, Fig. 3F) also ameliorated the negative affective bias at the 24 h timepoint, but did not induce a significant positive affective bias, although the *p* values for CP101,606 and ephenidine were less than *p* = 0.1.

**Fig. 4:**
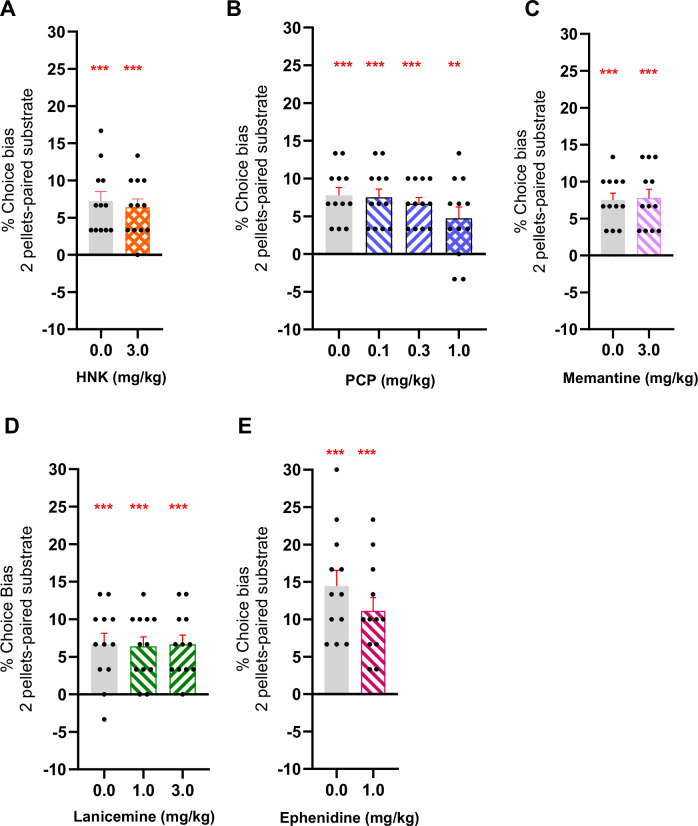
The reward learning assay has shown no general effects on memory retrieval. In the reward learning assay, a reward-induced positive bias was generated by using high (two pellets) versus low (one pellet) reward pairing sessions followed by administration of HNK (0.3, 1.0, 3.0 mg/kg; *n* = 12; **A**), PCP (0.1, 0.3, 1.0 mg/kg; *n* = 12; **B**), memantine (0.3, 1.0, 3.0 mg/kg; *n* = 12; **C**), lanicemine (1.0, 3.0, 10.0 mg/kg; *n* = 12, **D**) or ephenidine (1.0 mg/kg; *n* = 12, **E**) before the choice test. Data are shown as mean % choice bias ± SEM (bars) as well as individual data points (dots, *n* = 12). Data were analyzed with one sample t-test against a null hypothesis mean of 0% choice bias (**p < 0.01, ***p < 0.001).

Only the treatments where an acute modulation of affective bias had been observed were tested using the control, reward-learning assay. Animals treated with HNK, PCP, memantine, lanicemine and ephenidine developed similar reward-induced positive biases compared to the vehicle control, supporting specific affective bias modulation by the drug treatments (Fig. 4A–E). No general memory impairments were observed as determined in the reward learning assay.

There was no evidence of non-specific impairments for the NMDAR antagonist treatments tested during choice tests (Tables S4 - S6), apart from reward learning assay study with PCP treatment. Rats in the study with highest dose of PCP were significantly slower than vehicle group (Dunnett’s test, *p* < 0.001). In a few studies changes in latency to dig and number of trials to criterion during learning pairing sessions were observed (for details, see Tables S7 - S9). In the first week of acute effects of PCP, rats were slower to dig during pairing sessions with FG7142 comparing to the vehicle (paired t-test, t11 = 2.570, *p* = 0.0261) and in the last week of sustained effects of lanicemine study, rats were faster to dig during pairing sessions with FG7142 (paired t-test, t11 = 5.124, *p* = 0.0003). In the third week of acute effects of memantine study, rats did more trials to achieve criterion during pairing sessions with FG7142 (paired t-test, t14 = 2.987, *p* = 0.0098). The summary table (Table S10) with effect sizes (Cohen’s *d*) and 95% confidence intervals calculated from one sample t-test for the % choice bias across all experiments showed small to large effect sizes of treatments.

## Discussion

By comparing the neuropsychological effects of different NMDA antagonists in the ABT, and considering their clinical efficacy in MDD, these studies suggest the sustained modulation of negative affective biases is most relevant to antidepressant efficacy. Previous studies with ketamine found specific neuropsychological effects in rats following acute treatment [7, 8]. In the ABT, shortly after treatment (~1 h) negatively biased memories were retrieved with a neutral bias and 24 h post-treatment they are retrieved with a positive bias indicating ketamine can facilitate re-learning with a relatively more positive affective valence. Using the same ABT protocols we have also shown acute treatment with the muscarinic antagonist and RAAD scopolamine can attenuate negative biases but at 24 h this effect is only sustained at neutral and the re-learning effect with ketamine is not observed. In the current study, HNK, PCP, lanicemine and ephenidine but not CP101,606 or memantine, attenuated negative affective bias in rats during the acute effects of the drug. The lack of effects in the reward learning assay suggests this acute modulation was specific to affective bias modulation. When animals were tested 24 h after treatment only HNK induced a positive affective bias similar to ketamine. Lanicemine and ephenidine both showed sustained attenuation of the negative bias but with only a trend towards a re-learning effect and positive bias at 24 h. Interestingly, despite a lack of an acute effect, CP101,606 attenuated the negative bias at retrieval. Memantine had no effects in any of the ABT protocols used. Comparing the acute and sustained modulation of affective biases seen with these different compounds, the acute modulation of affective biases is less predictive of antidepressant efficacy than the sustained effects. The differences observed between the various NMDA antagonists tested suggests modulation of affective biases is sensitive to the drugs pharmacodynamic properties. Ephenidine is pharmacologically the most like ketamine and exhibits a similar profile of affective bias modification. This would also support the hypothesis that the NMDA receptor is the primary mediator of ketamine’s sustained antidepressant effects [28]. Ephenidine lacks affinity for opioid receptor [28, 29] and does not produce the metabolite HNK. Although HNK’s effects at higher doses were like ketamine’s in the ABT, the plasma concentrations achieved with an effective ketamine dose are likely to be much lower. HNK may have antidepressant effects but is unlikely to be the primary mediator of ketamine’s neuropsychological effects.

Behavioural studies using the forced swim test have suggested HNK could be the primary mediator of ketamine’s antidepressant effects. In the ABT, HNK attenuated negative biases at the higher doses tested (1.0–3.0 mg/kg) and induced a positive affective bias 24 h following treatment with the highest dose 3.0 mg/kg. However, since HNK required a higher dose than ketamine to achieve similar effects in the ABT, it is unlikely to be the primary mechanism thought which ketamine modulates affective biases. Studies using conventional models of depression suggest HNK interacts directly with AMPA receptors to mediate its antidepressant effects, but this has yet to be investigated in the translational ABT [20, 21]. Opioid receptors have also been proposed as mediators of ketamine’s antidepressant effects [36], however, the findings from this study showing the NMDA antagonist ephenidine replicates the effects of ketamine supports an NMDA mechanism. Ephenidine has similar pharmacodynamic properties as ketamine at the NMDA receptor but does not produce the active metabolite HNK in vivo or exhibit appreciably affinity for opioid receptors [28].

Both lanicemine and memantine have previously been investigated as antidepressants, however, neither has shown efficacy in clinical studies [18, 20, 37]. Memantine had no effects [37, 38] and while lanicemine showed some positive effects in early clinical trials, it was less potent than ketamine and with effects only lasting a few days [19]. Lanicemine has also been tested using emotional processing tasks in an experimental medicine study where it showed some positive effects [39] but later clinical trials failed to detect effects relative to placebo [18]. The profile of clinical effects with lanicemine, and its effects in the ABT are more similar to those seen with scopolamine, which also has some RAAD effects but with a shorter duration of action and requiring more frequent administration [8]. Lanicemine and memantine, are low-trapping or partial trapping and low potency NMDA channel blockers respectively [18, 20, 37]. Memantine had no effects in any of the ABT protocols, while lanicemine had acute effects at all doses tested but only sustained effects at the highest dose of 10 mg/kg. This dose likely generates higher plasma levels and receptor occupancy than clinical doses [18, 19] and at more clinically relevant doses, no sustained effects were observed. The range of doses we tested covered the highest end of human dose range and higher.

Divergence in the neuropsychological effects seen with the NMDA antagonists tested suggests their specific pharmacodynamic properties are important. We observed limited neuropsychological effects with the low/partial ion trapping and low potency NMDA antagonists with lanicemine having sustained effects only when given at the highest dose. When we tested the very high ion trapping and potent NMDA antagonists, PCP, we only observed acute effects with no difference from vehicle seen at 24 h post treatment supporting our hypothesis that pharmacodynamic properties are an important factor influencing antidepressant efficacy. Longer latencies following highest dose of PCP in the RLA indicate non-specific effects. These findings suggest the acute dissociative effects may have short term effects on the affective bias circuit but does not result in longer term modulation, or its more potent effects at the NMDA receptor limit specific modulation of the affective bias circuit.

To further explore this, we tested a NMDA antagonist with similar pharmacodynamic properties to ketamine, ephenidine. Both ketamine and ephenidine are relatively high ion trapping channel blockers at the NMDA receptor but lower than PCP [26]. They also act in a highly voltage-dependent manner [28, 40]. Ephenidine treatment led to both acute and sustained modulation of affective biases at a similar dose to ketamine, 1 mg/kg and there was a trend towards a re-learning mediated positive bias at 24 h, although only one dose of ephenidine was tested. Although these studies only looked at affective bias modification, the lack of affinity of ephenidine for opioid receptors suggests this is not the mediator of ketamine’s effects in the ABT and further supports a NMDA-mediated mechanism.

CP101,606 is a GluN2B subunit-selective NMDA receptor antagonist and has previously been shown to have positive effects in a clinical trial in treatment resistant depression [17]. Subsequent development of the drug, and larger scale clinical trials, have not been possible due to safety concerns associated with QT interval prolongation [41]. In the ABT, the effects of CP101,606 were different from the other NMDA antagonists tested with no acute effects observed but a sustained modulation of affective biases at 24 h. The sustained modulation of affective biases by CP101,606 suggests it is possible to dissociate between the acute and sustained modulation of affective biases and an acute effect is not necessary for the sustained effects to develop. The acute effects observed with the different NMDA antagonists tested suggest this may correspond with their acute dissociative effects, but these are not necessary for the antidepressant effects. Although safety concerns halted further development of CP101,606, the results from these ABT studies suggest NR2B selective antagonists can induce sustained modulation of affective biases without acute effects.

### Limitations and future directions

Our studies were subjected to a several limitations. As direct quantification of NMDA receptor occupancy was not included, we do not know if the dose ranges achieved similar levels of channel blockade. Although data for in vitro occupancy are known, the lack of direct in vivo occupancy data mean in vivo bioavailability, and the impact of metabolism and excretion on plasma and brain levels are not established. We also do not yet fully understand the relationship between the antidepressant effects of ketamine and NMDA receptor occupancy and how this relates to the dissociative effects. Our recent studies with ketamine suggest that higher doses of ketamine (>10 mg/kg) are not selective at modulating affective biases and instead induce more general behavioural impairments [8]. There are also different perspectives in the literature in terms of the extent to which the dissociative effects are important and contribute to the antidepressant effects [42]. It would be an interesting further characterisation of the compounds evaluated here to test additional lower doses and directly compare with respect to NMDA receptor occupancy, dissociative effects and affective bias modification. The findings from this study with CP101,606, suggest its acute effects differ from the other NMDA antagonists in terms of modulation of affective biases which we speculate could also reflect less dissociation. Further studies in rats using the different ABT protocols could be a useful way to further explore this question and the clinical relevance of these two important features of NMDA antagonism. Given this study utilised only male rats, we cannot directly address sex differences in responses to NMDA antagonists or make predictions about how this might impact on clinical effects. Prior work in female rats and across multiple strains has demonstrated a homological validity of the ABT and affective bias modification [5]. Nevertheless, future work comparing both sexes would be helpful to directly assess potential sex-dependent differences in NMDA antagonist efficacy. Future studies might also consider repeated ketamine dosing and longer observation periods. While the 24 h time point provides insight into sustained-acute effects, it does not fully replicate the repeated, intermittent dosing used clinically with ketamine as effects of an acute dose vary in terms of duration of effects from a few days to a couple of weeks [43]. Longitudinal sampling incorporating multiple post-dose time points, as well as repeated dosing paradigms, would allow a better characterisation of ketamine’s temporal profile and its relevance to clinical treatment schedules.

## Conclusions

These findings provide important insights into the role of NMDA antagonism in ketamine’s antidepressant effects and suggest pharmacodynamic properties may be important for efficacy. Findings in the ABT, and particularly the sustained modulation of negative affective biases, correspond well with clinical studies [1, 2] adding further to the hypothesis that modulation of affective biases is an important neuropsychological mechanism contributing to antidepressant efficacy. Building from these studies, sustained affective bias modulation by novel NMDA antagonists in the ABT may provide a predictive model to identify more effective therapeutic interventions with reduced side effects and abuse liability.

## Data and materials availability

All data are available in the main text or the supplementary materials. Individual-level data for all studies are available on the Open Science Framework at 10.17605/OSF.IO/MY8BN.

## Supplementary information

Supplementary materials
