# Supplementary material for: Differences in how NMDA antagonists modulate negative affective biases in male rats may serve as a predictor of clinical efficacy in major depressive disorder
Source: Transl Psychiatry. 2026 May 29;16:385. doi: 10.1038/s41398-026-04133-z (PMC13424567; doi:10.1038/s41398-026-04133-z)
Supplement: Supplementary file 1 — Supplementary materials [file 41398_2026_4133_MOESM1_ESM.docx]

**Supplementary Materials**

**Differences in how NMDA antagonists modulate negative affective biases in male rats may serve as a predictor of clinical efficacy in major depressive disorder**

Justyna K. Hinchcliffe^1^, Katie Kamenish^1^, Julia Bartlett^1^, Roberto Arban^2^, Bastian Hengerer^2^, Emma S.J. Robinson^1^*

**Affiliations:**

^1^ University of Bristol, School of Physiology, Pharmacology and Neuroscience, Biomedical Sciences Building, Bristol, BS8 1TD, UK

^2^ CNS Diseases Research, Boehringer Ingelheim GmbH & Co. KG, Biberach an der Riss, Germany

*Corresponding author email: [emma.s.j.robinson@bristol.ac.uk](mailto:emma.s.j.robinson@bristol.ac.uk)

**
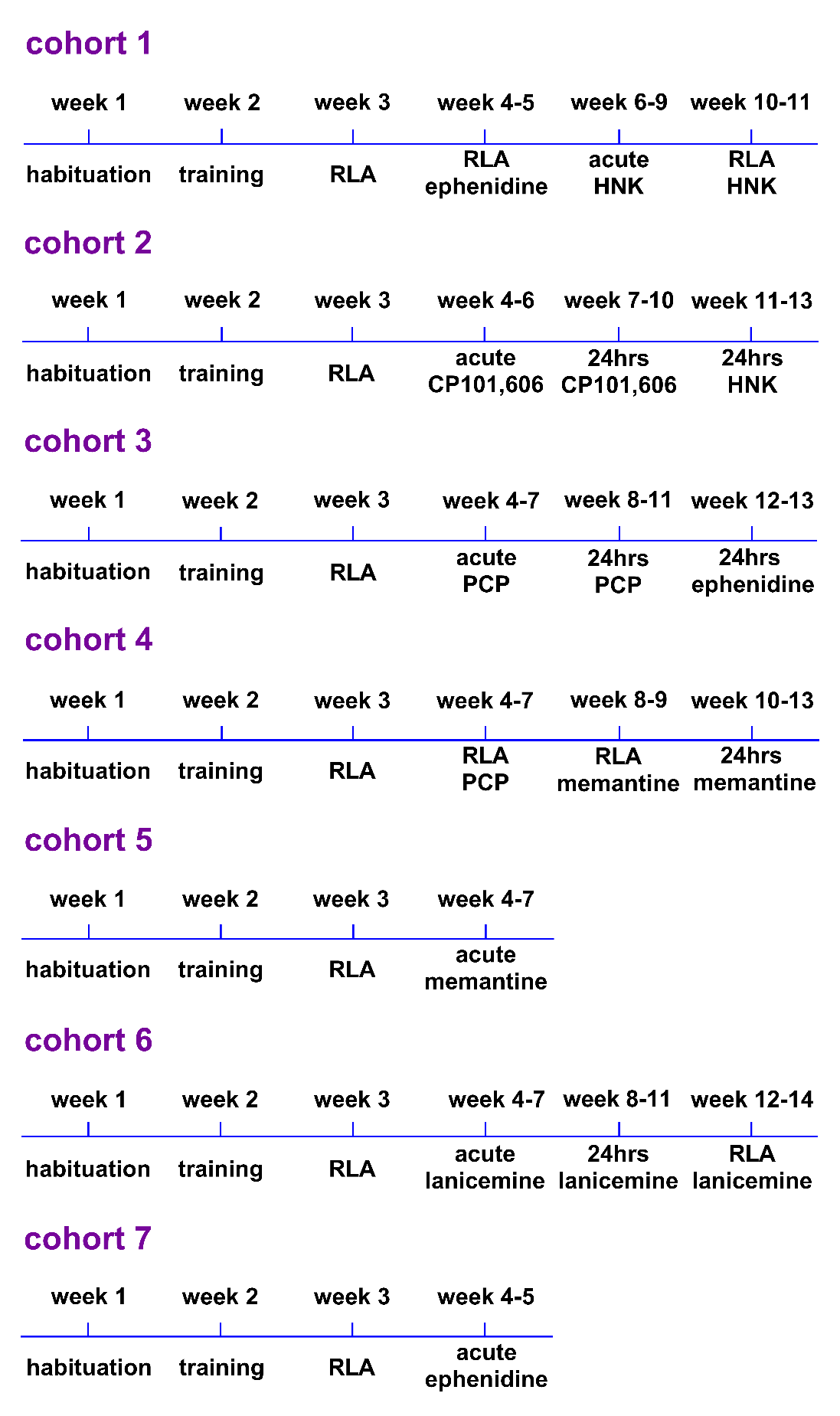
**

**Figure S1. Experimental timeline for all animal cohorts used.**

| Cohort | Treatment | Dose (mg/kg) | Route of administration | Pre-treatment times |
| --- | --- | --- | --- | --- |
| 1, 2 | HNK | 0.0, 0.3, 1.0, 3.0 | IP (systemic) | 20min./24hrs |
| 3, 4 | PCP | 0.0, 0.1, 0.3, 1.0 | IP (systemic) | 40min./24hrs |
| 4, 5 | Memantine | 0.0, 0.3, 1.0, 3.0 | IP (systemic) | 60min./24hrs |
| 6 | Lanicemine | 0.0, 1.0, 3.0, 10.0 | IP (systemic) | 60min./24hrs |
| 2 | CP101,606 | 0.0, 1.0, 3.0 | IP (systemic) | 60min./24hrs |
| 1, 3, 7 | Ephenidine | 0.0, 1.0 | IP (systemic) | 60min./24hrs |
| 3, 7 | Corticosterone | 0.0, 10.0 | SC (systemic) | 30min. |
| 1, 2, 4, 5, 6 | FG7142 | 0.0, 3.0 | SC (systemic) | 30min. |

**Table S1: Summary of drug treatments in all animal cohorts.**

|  | Day 1 | Day 2 | Day 3 | Day 4 | Day 5 | Day 5/6 |
| --- | --- | --- | --- | --- | --- | --- |
|  | **Pairing 1** | **Pairing 2** | **Pairing 3** | **Pairing 4** | **Treatment with NMDAR antagonist** | **Choice Test** |
| Group 1 | CS+A vs. CS-  **Drug** | CS+B vs. CS-  **Vehicle** | CS+A vs. CS-  **Drug** | CS+B vs. CS-  **Vehicle** | **Drug A**  acute or 24hrs prior | CS+A vs. CS+B,  30 trials |
| Group 2 | CS+B vs. CS-  **Drug** | CS+A vs. CS-  **Vehicle** | CS+B vs. CS-  **Drug** | CS+A vs. CS-  **Vehicle** | **Drug B**  acute or 24hrs prior | CS+A vs. CS+B,  30 trials |
| Group 3 | CS+A vs. CS-  **Vehicle** | CS+B vs. CS-  **Drug** | CS+A vs. CS-  **Vehicle** | CS+B vs. CS-  **Drug** | **Drug C**  acute or 24hrs prior | CS+A vs. CS+B,  30 trials |
| Group 4 | CS+B vs. CS-  **Vehicle** | CS+A vs. CS-  **Drug** | CS+B vs. CS-  **Vehicle** | CS+A vs. CS-  **Drug** | **Drug D**  acute or 24hrs prior | CS+A vs. CS+B,  30 trials |

**Table S2A: Standard procedure for testing drug-induced affective bias versus vehicle.** Each animal receives drug treatment (Drug, i.e. corticosterone or FG7142) or vehicle counterbalanced over the four substrate-reward pairing sessions to induce a negative affective bias. On day 5 or 6 they undergo treatment with NMDAR antagonist (e.g. Drug A, B, C, D) either acutely or 24hrs prior to choice test. Substrate and day are also counter-balanced resulting in four different groups.

|  | Day 1 | Day 2 | Day 3 | Day 4 | Day 5 |
| --- | --- | --- | --- | --- | --- |
|  | **Pairing 1** | **Pairing 2** | **Pairing 3** | **Pairing 4** | **Choice Test with prior treatment with NMDAR antagonist** |
| Group 1 | CS+A vs. CS-  **2 pellets** | CS+B vs. CS-  **1 pellet** | CS+A vs. CS-  **2 pellets** | CS+B vs. CS-  **1 pellet** | **Drug A**  Choice test  CS+A vs. CS+B |
| Group 2 | CS+B vs. CS-  **2 pellets** | CS+A vs. CS-  **1 pellet** | CS+B vs. CS-  **2 pellets** | CS+A vs. CS-  **1 pellet** | **Drug B**  Choice test  CS+A vs. CS+B |
| Group 3 | CS+A vs. CS-  **1 pellet** | CS+B vs. CS-  **2 pellets** | CS+A vs. CS-  **1 pellet** | CS+B vs. CS-  **2 pellets** | **Drug C**  Choice test  CS+A vs. CS+B |
| Group 4 | CS+B vs. CS-  **1 pellet** | CS+A vs. CS-  **2 pellets** | CS+B vs. CS-  **1 pellet** | CS+A vs. CS-  **2 pellets** | **Drug D**  Choice test  CS+A vs. CS+B |

**Table S2B: Standard procedure for testing in the reward learning assay***.* Each animal receives 2 pellets or 1 pellet counterbalanced over the four substrate-reward pairing sessions, following the NMDAR antagonist treatment (e.g. Drug A, B, C, D) prior to choice test on day 5. Substrate and day are also counter-balanced resulting in four different groups.

| **Study** | **Number of rats excluded** |
| --- | --- |
| Corticosterone-induced negative bias and PCP 0.1, 0.3, 1.0mg/kg at recall 24h | 2 (1- more than 2 SDs, 1- positive choice bias in the vehicle group) |
| Corticosterone-induced negative bias and ephenidine 1.0mg/kg at recall 1h | 2 (1- more than 2 SDs, 1- positive choice bias in the vehicle group) |
| FG7142-induced negative bias and CP101606 1.0, 3.0mg/kg at recall 1h | 2 (positive choice bias in the vehicle group) |
| FG7142-induced negative bias and CP101,606 1.0mg/kg at recall 24h | 1 (positive choice bias in the vehicle group) |
| FG71421-induced negative bias and CP101,606 3.0mg/kg at recall 24h | 1 (positive choice bias in the vehicle group) |
| Reward learning assay with memantine at recall 1h | 1 (more than 2 SDs) |

**Table S3: Summary of data exclusions.**

| **Treatment** | **Dose (mg/kg)** | **Response latency (s)** |
| --- | --- | --- |
| Hydroxynorketamine | 0.0 | 2.5±0.2 |
|  | 0.3 | 2.8±0.4 |
|  | 1.0 | 2.4±0.2 |
|  | 3.0 | 2.7±0.3 |
| Ephenidine | 0 | 3.1±0.3 |
|  | 1 | 3.0±0.4 |
| PCP | 0 | 2.4±0.2 |
|  | 0.1 | 1.9±0.1 |
|  | 0.3 | 2.2±0.2 |
|  | 1 | 1.8±0.1 |
| CP101,606 | 0 | 3.5 ± 0.3 |
|  | 1 | 2.9 ± 0.2 |
|  | 3 | 3.2 ± 0.4 |
| Memantine | 0 | 1.8±0.1 |
|  | 0.3 | 1.8±0.1 |
|  | 1 | 1.7±0.1 |
|  | 3 | 2.4±0.5 |
| Lanicemine | 0 | 1.4±0.1 |
|  | 1 | 1.4±0.1 |
|  | 3 | 1.5±0.1 |
|  | 10 | 1.5±0.1 |

**Table S4: Choice bias data: response latency to dig in the acute modulation studies.** Data shown as mean ± SEM (n=10-12 animals/group). No significant difference in latency to make choice was observed in studies following treatment with vehicle or any of the drugs: hydroxynorketamine, ephenidine, PCP, CP101,606, memantine and lanicemine.

| **Treatment** | **Dose (mg/kg)** | **Response latency (s)** |
| --- | --- | --- |
| Hydroxynorketamine | 0.0 | 4.1 ± 0.5 |
|  | 1.0 | 3.9 ± 0.7 |
|  | 3.0 | 4.3 ± 0.7 |
| Ephenidine | 0 | 2.0±0.1 |
|  | 0.1 | 1.8±0.0 |
| PCP | 0 | 2.3±0.1 |
|  | 0.1 | 2.3±0.1 |
|  | 0.3 | 2.3±0.1 |
|  | 1 | 2.2±0.1 |
| CP101,606 | 0 | 3.8 ± 0.4 |
|  | 1 | 5.5 ± 1.1 |
|  | 0 | 3.9 ± 0.5 |
|  | 3 | 4.7 ± 1.1 |
| Memantine | 0 | 1.5±0.1 |
|  | 0.3 | 1.4±0.0 |
|  | 1 | 1.5±0.0 |
|  | 3 | 1.6±0.1 |
| Lanicemine | 0 | 1.6±0.1 |
|  | 1 | 1.6±0.1 |
|  | 3 | 1.6±0.1 |
|  | 10 | 1.5±0.1 |

**Table S5: Choice bias data: response latency to dig latency to dig in the sustained modulation studies.** Data shown as mean ± SEM (n=10-12 animals/group). No significant difference in latency to make choice was observed in studies following treatment with vehicle or any of the drugs: hydroxynorketamine, ephenidine, PCP, CP101,606, memantine and lanicemine.

| **Treatment** | **Dose (mg/kg)** | **Response latency (s)** |
| --- | --- | --- |
| Hydroxynorketamine | **0.0** | **3.5±0.3** |
|  | 3.0 | 3.2±0.2 |
| Ephenidine | 0 | 2.6±0.1 |
|  | 0.1 | 2.4±0.2 |
| PCP | 0 | **1.6±0.0** |
|  | 0.1 | **1.7±0.1** |
|  | 0.3 | **1.6±0.1** |
|  | 1 | **3.8±0.3***** |
| Memantine | 0 | 1.4±0.0 |
|  | 3 | 1.4±0.0 |
| Lanicemine | 0 | 1.5±0.1 |
|  | 1 | 1.5±0.0 |
|  | 3 | 1.5±0.0 |

**Table S6: Choice test data: response latency to dig in the reward learning assay.** Data shown as mean ± SEM (n=11-12 animals/group). The only significant difference was observed in the PCP (0.1-1.0mg/kg) study (RM ANOVA, F(3, 33) = 50.04, p<0.0001), rats were significantly slower to make a choice following highest dose (1mg/kg, p<0.0001) of PCP comparing to vehicle treatment.

| **Treatment** |  | **Response latency (s)** | | **Trials to criterion** | |
| --- | --- | --- | --- | --- | --- |
|  |  | **Vehicle** | **Drug** | **Vehicle** | **Drug** |
| Hydroxynorketamine | Week 1 | 3.3±0.4 | 3.2±0.3 | 6.5±0.2 | 6.7±0.3 |
|  | Week 2 | 3.1±0.4 | 3.3±0.5 | 6.3±0.2 | 6.1±0.1 |
|  | Week 3 | 3.4±0.5 | 3.1±0.3 | 6.5±0.3 | 6.7±0.3 |
|  | Week 4 | 2.8±0.4 | 2.7±0.3 | 6.4±0.3 | 6.0±0.0 |
| Ephenidine | Week 1 | 3.7±0.5 | 3.8±0.5 | 6.4±0.2 | 6.2±0.1 |
|  | Week 2 | 3.1±0.2 | 3.8±0.5 | 6.2±0.1 | 6.3±0.2 |
| PCP | Week 1 | **3.7±0.3** | **4.7±0.6***** | 6.8±0.1 | 6.7±0.1 |
|  | Week 2 | 2.7±0.1 | 2.8±0.2 | 6.3±0.1 | 6.3±0.1 |
|  | Week 3 | 2.4±0.1 | 2.4±0.1 | 6.3±0.1 | 6.2±0.1 |
|  | Week 4 | 2.3±0.1 | 2.3±0.2 | 6.3±0.1 | 6.3±0.1 |
| CP101,606 | Week 1 | 4.1±0.4 | 4.7±0.7 | 7.3±0.5 | 7.9±0.5 |
|  | Week 2 | 3.9±0.4 | 4.2±0.5 | 7.0±0.3 | 7.6±0.4 |
|  | Week 3 | 4.2±0.6 | 3.6±0.4 | 6.4±0.2 | 6.1±0.1 |
| Memantine | Week 1 | 1.6±0.1 | 2.0±0.0 | 6.3±0.2 | 6.3±0.2 |
|  | Week 2 | 1.8±0.1 | 1.9±0.1 | 6.3±0.2 | 6.8±0.2 |
|  | Week 3 | 1.8±0.1 | 2.1±0.1 | **6.8±0.3** | **8.0±0.3***** |
|  | Week 4 | 1.8±0.1 | 2.0±0.1 | 6.7±0.3 | 7.0±0.2 |
| Lanicemine | Week 1 | 1.5±0.0 | 1.6±0.2 | 8.5±0.7 | 9.3±0.6 |
|  | Week 2 | 1.6±0.1 | 1.6±0.0 | 7.5±0.5 | 7.1±0.4 |
|  | Week 3 | 1.6±0.1 | 1.6±0.1 | 7.8±0.5 | 7.0±0.3 |
|  | Week 4 | 1.4±0.1 | 1.4±0.1 | 7.1±0.4 | 7.2±0.3 |

**Table S7:** **Pairing sessions data: number of trials to criterion and latency to dig in the acute modulation studies**. Data shown as mean ± SEM (n=10-12 animals/group) averaged from the two pairing sessions for each substrate-reward association (vehicle or drug). There were no significant effects during pairing sessions, either on response latency to dig or number of trials to criterion following treatment with vehicle or any of the drugs. Only during first week of PCP study we observed difference in response latency (paired t-test, t11=2.570, p=0.0261) resulted in slower latency to dig during pairing sessions with FG7142 comparing to the vehicle and during third week of memantine we observed difference in trials to criterion (paired t-test, t14=2.987, p=0.0098) resulted in rats doing more trials to achieve criterion during pairing sessions with FG7142.

| **Treatment** |  | **Response latency (s)** | | **Trials to criterion** | |
| --- | --- | --- | --- | --- | --- |
|  |  | **Vehicle** | **Drug** | **Vehicle** | **Drug** |
| Hydroxynorketamine | Week 1 | 3.4±0.4 | 3.7±0.4 | 6.6±0.2 | 7.2±0.4 |
|  | Week 2 | 3.7±0.3 | 3.6±0.3 | 6.6±0.2 | 7.1±0.4 |
|  | Week 3 | 3.0±0.2 | 2.9±0.2 | 6.8±0.2 | 6.5±0.3 |
| Ephenidine | Week 1 | 2.4±0.1 | 2.2±0.1 | 6.4±0.1 | 6.4±0.1 |
|  | Week 2 | 2.3±0.2 | 2.2±0.1 | 6.3±0.1 | 6.4±0.1 |
| PCP | Week 1 | 2.4±0.1 | 2.6±0.2 | 6.5±0.1 | 6.6±0.1 |
|  | Week 2 | 2.7±0.1 | 2.7±0.1 | 6.3±0.1 | 6.3±0.1 |
|  | Week 3 | 2.6±0.1 | 2.5±0.2 | 7.0±0.3 | 7.0±0.2 |
|  | Week 4 | 2.4±0.2 | 2.2±0.1 | 6.5±0.1 | 6.3±0.1 |
| CP101,606 1.0mg/kg | Week 1 | 3.7±0.5 | 3.9±0.6 | 6.0±0.0 | 6.0±0.0 |
|  | Week 2 | 4.6±0.6 | 4.9±0.7 | 6.7±0.3 | 7.0±0.3 |
| CP101,606 3.0mg/kg | Week 1 | 3.1±0.3 | 3.0±0.3 | 6.5±0.3 | 6.0±0.0 |
|  | Week 2 | 4.1±0.4 | 4.2±0.5 | 6.6±0.4 | 6.0±0.0 |
| Memantine | Week 1 | 1.5±0.0 | 1.5±0.0 | 8.6±0.5 | 7.5±0.2 |
|  | Week 2 | 1.6±0.0 | 1.5±0.1 | 8.4±0.5 | 7.2±0.3 |
|  | Week 3 | 1.5±0.0 | 1.5±0.1 | 8.8±0.4 | 7.6±0.2 |
|  | Week 4 | 1.5±0.0 | 1.5±0.1 | 8.6±0.6 | 7.4±0.3 |
| Lanicemine | Week 1 | 1.6±0.0 | 1.7±0.0 | 9.5±0.3 | 9.4±0.3 |
|  | Week 2 | 1.5±0.0 | 1.5±0.1 | 9.2±0.5 | 9.2±0.3 |
|  | Week 3 | 1.7±0.1 | 1.6±0.1 | 8.7±0.4 | 9.2±0.3 |
|  | Week 4 | **1.6±0.0** | **1.4±0.0***** | 8.1±0.2 | 8.9±0.3 |

**Table S8: Pairing sessions data: number of trials to criterion and latency to dig in the sustained modulation studies.** Data shown as mean ± SEM (n=10-12 animals/group) averaged from the two pairing sessions for each substrate-reward association (vehicle or drug). There were no significant effects during pairing sessions, either on response latency to dig or number of trials to criterion following treatment with vehicle or any of the drugs. Only during last week of lanicemine study we observed difference in response latency (paired t-test, t11=5.124, p=0.0003) resulted in faster latency to dig during pairing sessions with FG7142 comparing to the vehicle.

| **Treatment** |  | **Response latency (s)** | | **Trials to criterion** | |
| --- | --- | --- | --- | --- | --- |
|  |  | **1 pellet** | **2 pellets** | **1 pellet** | **2 pellets** |
| Hydroxynorketamine | Week 1 | 4.0±0.5 | 3.3±0.3 | 6.5±0.2 | 6.7±0.2 |
|  | Week 2 | 3.5±0.3 | 3.1±0.2 | 6.5±0.2 | 6.4±0.2 |
| Ephenidine | Week 1 | 4.0±0.4 | 3.7±0.3 | 6.6±0.3 | 7.3±0.4 |
|  | Week 2 | 2.6±0.2 | 2.9±0.2 | 7.0±0.4 | 6.7±0.2 |
| PCP | Week 1 | 1.6±0.1 | 1.9±0.1 | 6.8±0.2 | 7.3±0.4 |
|  | Week 2 | 1.8±0.1 | 1.9±0.1 | 7.5±0.3 | 7.9±0.4 |
|  | Week 3 | 1.6±0.1 | 1.8±0.1 | 7.5±0.3 | 8.3±0.4 |
|  | Week 4 | 1.6±0.0 | 1.8±0.1 | 7.3±0.3 | 8.6±0.4 |
| Memantine | Week 1 | 1.5±0.0 | 1.6±0.1 | 7.8±0.2 | 7.6±0.3 |
|  | Week 2 | 1.5±0.0 | 1.5±0.1 | 6.9±0.2 | 7.8±0.5 |
| Lanicemine | Week 1 | **1.7±0.1** | **1.5±0.0***** | 9.3±0.4 | 8.3±0.3 |
|  | Week 2 | 1.6±0.0 | 1.5±0.0 | 9.3±0.4 | 8.4±0.3 |
|  | Week 3 | 1.7±0.1 | 1.7±0.0 | 8.3±0.3 | 7.9±0.2 |

**Table S9: Pairing sessions data: number of trials to criterion and latency to dig in the reward learning assay.** Data shown as mean ± SEM (n=11-12 animals/group) averaged from the two pairing sessions for each substrate-reward association (1 pellet or 2 pellets). There were no significant effects during pairing sessions, either on response latency to dig or number of trials to criterion. Only during first week of lanicemine study we observed difference in response latency (paired t-test, t11=2.499, p=0.0296) resulted in faster latency to dig during pairing sessions with 1 pellet comparing to the 2 pellets.

| **Treatment and dose (mg/kg)** | | **Cohen’s d** | **95% confidence intervals** | **Treatment and dose (mg/kg)** | | **Cohen’s d** | **95% confidence intervals** |
| --- | --- | --- | --- | --- | --- | --- | --- |
| **Figure 2A**  **HNK** | **0.0** | 2.49 | -12.90 to -7.651 | **Figure 2B**  **PCP** | **0.0** | 2.85 | -11.21 to -7.122 |
|  | **0.3** | 0.82 | -8.386 to -1.059 |  | **0.1** | 0.41 | -5.641 to 1.197 |
|  | **1.0** | 0.48 | -7.140 to 1.029 |  | **0.3** | 0.53 | -5.512 to 0.5121 |
|  | **3.0** | 0.44 | -6.126 to 1.126 |  | **1.0** | 0.46 | -3.296 to 0.5179 |
| **Figure 2C**  **Memantine** | **0.0** | 1.68 | -8.908 to -3.819 | **Figure 2D**  **Lanicemine** | **0.0** | 1.85 | -10.07 to -4.926 |
|  | **0.3** | 1.57 | -8.229 to -3.286 |  | **1.0** | 0.39 | -5.872 to 1.428 |
|  | **1.0** | 1.75 | -10.07 to -4.473 |  | **3.0** | 0.13 | -3.317 to 4.984 |
|  | **3.0** | 0.82 | -10.20 to -1.009 |  | **10.0** | 0.76 | 0.6537 to 7.124 |
| **Figure 2E**  **CP101,606** | **0.0** | 1.46 | -12.43 to -4.242 | **Figure 2F**  **Ephenidine** | **0.0** | 1.36 | -8.155 to -2.754 |
|  | **1.0** | 0.47 | -10.89 to 2.226 |  | **1.0** | 0.27 | -3.169 to 1.351 |
|  | **3.0** | 0.80 | -13.87 to -0.7982 |  |  |  |  |
| **Figure 3A**  **HNK** | **0.0** | 1.21 | -10.16 to -3.169 | **Figure 3B**  **PCP** | **0.0** | 2.25 | -12.73 to -6.599 |
|  | **1.0** | 0.08 | -4.122 to 5.234 |  | **0.1** | 1.73 | -8.958 to -3.709 |
|  | **3.0** | 0.84 | 1.022 to 7.311 |  | **0.3** | 1.91 | -9.624 to -4.376 |
|  |  |  |  |  | **1** | 1.36 | -8.651 to -2.682 |
| **Figure 3C**  **Memantine** | **0.0** | 2.09 | -9.054 to -4.835 | **Figure 3D**  **Lanicemine** | **0.0** | 1.90 | -10.38 to -5.171 |
|  | **0.3** | 1.79 | -9.411 to -4.478 |  | **1.0** | 1.71 | -9.906 to -4.538 |
|  | **1.0** | 2.30 | -10.64 to -6.031 |  | **3.0** | 1.13 | -9.105 to -2.562 |
|  | **3.0** | 2.43 | -10.17 to -5.946 |  | **10.0** | 0.05 | -4.158 to 3.602 |
| **Figure 3E**  **CP101,606** | **0.0** | 1.87 | -18.14 to -8.530 | **Figure 3F**  **Ephenidine** | **0.0** | 1.98 | -9.911 to -5.089 |
|  | **1.0** | 0.06 | -7.815 to 6.603 |  | **1.0** | 0.55 | -0.3736 to 5.374 |
|  | **0.0** | 1.22 | -8.939 to -2.576 |  |  |  |  |
|  | **3.0** | 0.56 | -0.8730 to 9.358 |  |  |  |  |
| **Figure 4A**  **HNK** | **0.0** | 1.62 | 4.390 to 10.05 | **Figure 4B**  **PCP** | **0** | 2.17 | 5.505 to 10.05 |
|  | **3.0** | 1.65 | 3.923 to 8.855 |  | **0.1** | 1.98 | 5.089 to 9.911 |
|  |  |  |  |  | **0.3** | 2.35 | 4.861 to 8.473 |
|  |  |  |  |  | **1.0** | 0.91 | 1.409 to 8.035 |
| **Figure 4C**  **Memantine** | **0.0** | 2.33 | 5.456 to 9.544 | **Figure 4D**  **Lanicemine** | **0.0** | 1.30 | 3.411 to 9.923 |
|  | **3.0** | 1.90 | 5.171 to 10.38 |  | **1.0** | 1.46 | 3.612 to 9.166 |
|  |  |  |  |  | **3.0** | 1.56 | 3.957 to 9.376 |
| **Figure 4E**  **Ephenidine** | **0.0** | 1.98 | 9.810 to 19.08 |  |  |  |  |
|  | **1.0** | 1.78 | 7.140 to 15.08 |  |  |  |  |

**Table S10.** **Summary of effect sizes (Cohen’s d) and 95% confidence intervals for the % choice bias across all experiments calculated from one sample t-test.**
